# Meiosis Progression and Recombination in Holocentric Plants: What Is Known?

**DOI:** 10.3389/fpls.2021.658296

**Published:** 2021-04-22

**Authors:** Paulo G. Hofstatter, Gokilavani Thangavel, Marco Castellani, André Marques

**Affiliations:** Department of Chromosome Biology, Max Planck Institute for Plant Breeding Research, Cologne, Germany

**Keywords:** holocentric chromosome, meiotic recombination, cohesion, centromere, inverted meiosis

## Abstract

Differently from the common monocentric organization of eukaryotic chromosomes, the so-called holocentric chromosomes present many centromeric regions along their length. This chromosomal organization can be found in animal and plant lineages, whose distribution suggests that it has evolved independently several times. Holocentric chromosomes present an advantage: even broken chromosome parts can be correctly segregated upon cell division. However, the evolution of holocentricity brought about consequences to nuclear processes and several adaptations are necessary to cope with this new organization. Centromeres of monocentric chromosomes are involved in a two-step cohesion release during meiosis. To deal with that holocentric lineages developed different adaptations, like the chromosome remodeling strategy in *Caenorhabditis elegans* or the inverted meiosis in plants. Furthermore, the frequency of recombination at or around centromeres is normally very low and the presence of centromeric regions throughout the entire length of the chromosomes could potentially pose a problem for recombination in holocentric organisms. However, meiotic recombination happens, with exceptions, in those lineages in spite of their holocentric organization suggesting that the role of centromere as recombination suppressor might be altered in these lineages. Most of the available information about adaptations to meiosis in holocentric organisms is derived from the animal model *C. elegans*. As holocentricity evolved independently in different lineages, adaptations observed in *C. elegans* probably do not apply to other lineages and very limited research is available for holocentric plants. Currently, we still lack a holocentric model for plants, but good candidates may be found among Cyperaceae, a large angiosperm family. Besides holocentricity, chiasmatic and achiasmatic inverted meiosis are found in the family. Here, we introduce the main concepts of meiotic constraints and adaptations with special focus in meiosis progression and recombination in holocentric plants. Finally, we present the main challenges and perspectives for future research in the field of chromosome biology and meiosis in holocentric plants.

## Introduction

### Meiosis, Conserved Mechanisms and Adaptations

Meiosis is a type of cell division responsible for reducing the number of chromosomes in diploid cells by half to produce haploid cells. It is a central step responsible for shuffling genetic information through meiotic recombination and produce genetic variation in eukaryotic life-cycles (Zickler and Kleckner, 2015). This is possible due to two rounds of cell division after a single DNA replication event with the participation of a specific and specialized meiotic machinery (Schurko and Logsdon, 2008).

Preliminary evidence suggests that meiosis is an ancestral feature of eukaryotes, what can robustly explain the patterns of pervasive occurrence of sexual processes in all eukaryotic diversity (Speijer et al., 2015). Despite the extreme conservation of the main meiotic steps even in the most distantly related groups, several lineages have specific meiotic adaptations. In *Drosophila*, several components of the core eukaryotic machinery playing roles in meiosis have been lost or even replaced: the meiosis-specific DMC1 recombinase was replaced by a distant homolog of it, spin-D/RAD51C (Abdu et al., 2003). *Schizosaccharomyces pombe* has lost the main meiotic pathway to resolve crossovers (COs) and heavily relies on a secondary pathway for the resolution of COs (which lacks interference) (Cromie et al., 2006). As a result, CO numbers are significantly higher in *S. pombe* compared to other model organisms, such as *Arabidopsis thaliana* (this plant presents around 1.5 CO per bivalent and both crossover resolution pathways are present) (Mercier et al., 2015). *S. pombe* has also lost the synaptonemal complex (Lorenz et al., 2004) and, thus, performs meiosis with a highly reduced machinery when compared to other well-characterized models. However, meiotic specializations are not restricted to the molecular machinery underpinning the main steps of the process. Some organisms exhibit morphological specializations as a consequence of structural peculiarities of chromosomal organization. For instance, homologous chromosomes (homologs) from some species of the genus *Oenothera* do not synapse upon meiosis rendering them functionally asexual even though they perform meiotic divisions (Johnson et al., 2009). This is due to large scale rearrangements inside the chromosomes, what leads to a state of permanent translocation heterozygosity. Another challenge to the regular progression of meiosis is the evolution of holocentric chromosomes in several lineages. In the main holocentric model, the nematode *C. elegans*, meiosis progresses in such a way that only a single chiasma is formed for each chromosome pair (Martinez-Perez et al., 2008; Martinez-Perez and Colaiacovo, 2009). In the case of holocentric plants of the families *Cyperaceae* (sedges) and Juncaceae (rushes), inverted meiosis evolved to cope with the holocentric chromosome structure: sister-chromatids are separated in the first meiotic division, while homologs are separated only upon the second division (Cabral et al., 2014; Heckmann et al., 2014; Marques et al., 2016). In an even more extreme case, *Rhynchospora tenuis* (Cyperaceae) presents achiasmatic inverted meiosis, whose viability seems to be possible due to the very small number of holocentric chromosomes inside the nucleus (just two pairs) so that even random segregation would produce some viable offspring (Cabral et al., 2014).

### Meiosis Progression and Recombination in Monocentric Plants

**Figure 1:**
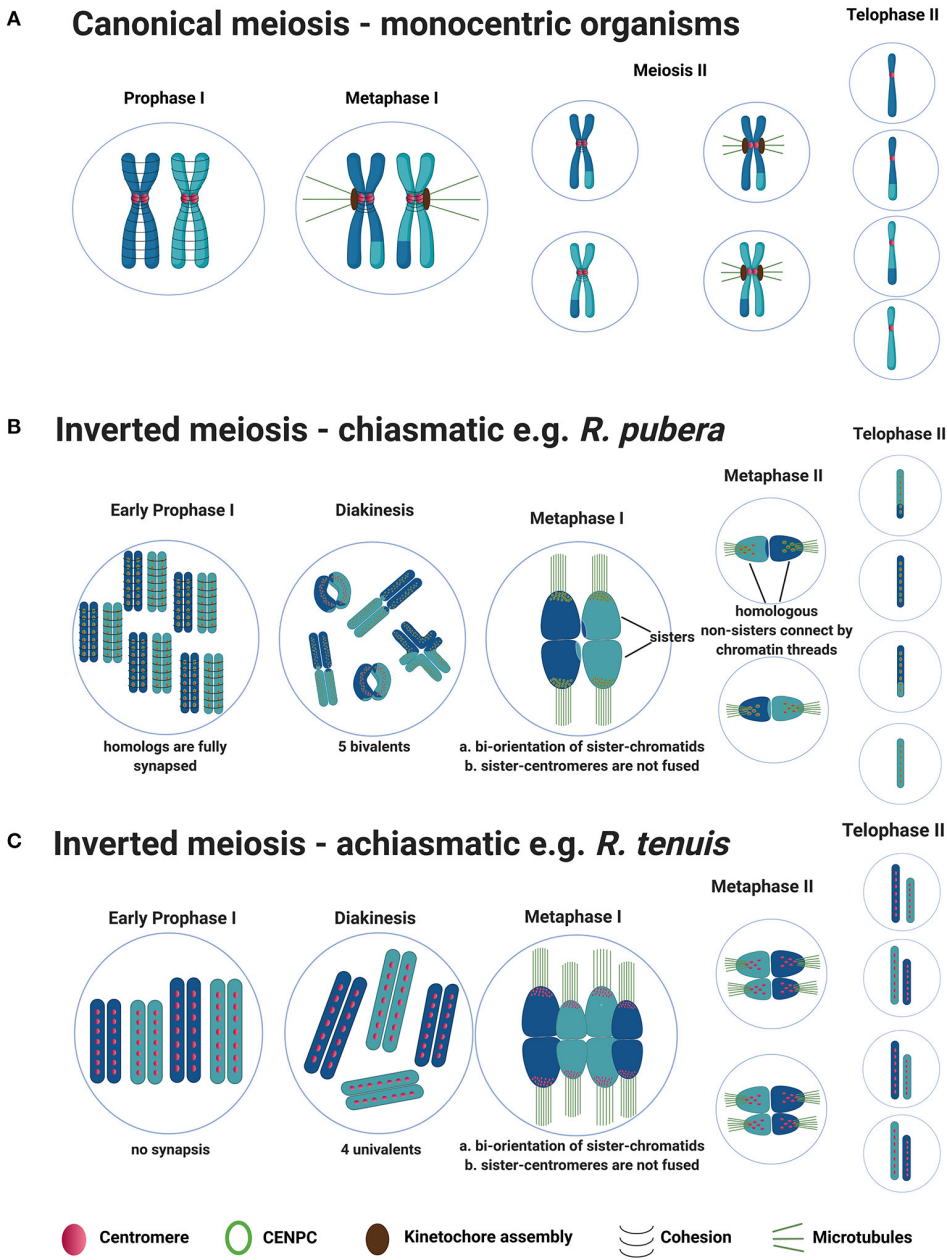
General model for canonical meiosis in monocentric organisms vs. inverted meiosis (both chiasmatic and achiamatic) in holocentric plants. **(A)** Canonical meiosis: During meiosis I reciprocal genetic exchange between homologs (crossovers) occurs, sisters-chromatids mono-orient via fused sister-centromeres and segregate to the same poles. During meiosis II, sisters-chromatids bi-orient and segregate to the opposite poles, resulting in four haploid gametes at the end. **(B)** Schematic representation of chiasmatic inverted meiosis observed in *R. pubera* (from metaphase I only one bivalent is illustrated for better understanding). During meiosis I, COs take place but the difference is that, centromeres from sisters are not fused, sister chromatids bi-orient and segregate to the opposite poles already at anaphase I. During meiosis II homologous non-sisters align, bi-orient and segregate to the opposite poles, resulting in four haploid gametes similar to canonical meiosis. **(C)** Schematic representation of achiasmatic inverted meiosis observed in *R. tenuis*. The sequence of events during inverted meiosis observed in *R. tenuis* is similar to that of *R. pubera*, but meiosis in *R. tenuis* is reported to be achiasmatic i.e., crossover formation doesn't occur during prophase I. As a result, four univalents are observed during diakinesis instead of two bivalents.

The sequence of events associated with canonical (monocentric) meiosis is well-established (Figure 1A). The homologs pair and synapse by the formation of the synaptonemal complex. After the introduction of double-strand breaks onto DNA, a process of DNA repair based on inter-homolog recombination ensues (Zickler and Kleckner, 2015). The sister chromatids are held together by cohesion along the chromosome arms and centromeres. By the end of prophase I, the homologs have recombined, are physically connected by chiasmata, and meiotic cohesin REC8 along chromosome arms is released (Xu et al., 2005). This segregation scheme necessitates a two-step loss of sister chromatid cohesion. Cohesin is removed distally to chiasmata to allow homologs to segregate during meiosis I while being partially maintained to enable sister-chromatids to partition correctly during meiosis II. In organisms that are monocentric, this sequential loss of cohesion is regulated by shugoshin which is specifically associated to centromeres (Kitajima et al., 2004). Shugoshin protects cohesin at the centromere until meiosis II by recruiting the conserved phosphatase, PP2A, to antagonize the phosphorylation and removal of the cohesin complex (Kitajima et al., 2006; Riedel et al., 2006). At metaphase I, the bivalents align to the metaphase plate with the sister kinetochores being poleward mono-oriented. At anaphase I, homologous centromeres are bi-oriented, the bivalents are detached, as chiasmata are resolved, and the homologs migrate to opposite poles. The sisters are held together until metaphase II by centromeric cohesion. The sister kinetochores now face opposite poles during metaphase II, centromeric cohesion is lost, the sister-chromatids are released and migrate to opposite poles as well. At the end of the meiosis, each nucleus has a haploid number of chromosomes (Mercier et al., 2015).

Meiotic recombination is essential to sexual reproduction and the generation of genetic diversity and, thus, has a profound effect on patterns of genetic variation and is an important tool for crop breeding (Taagen et al., 2020). Variation in recombination rates is of particular interest due to efforts to increase the rate of genetic gain in agricultural crops by breaking up large linkage blocks containing both beneficial and detrimental alleles. Meiotic recombination events (crossovers, i.e., COs) are unevenly distributed throughout eukaryotic genomes, some regions exhibiting higher recombination rates (hotspots), while other exhibiting lower rates (cold spots) (Petes, 2001; Fernandes et al., 2019). The causes of this observed uneven distribution are currently not well-understood.

In most eukaryotes there is at least one CO per chromosome per meiotic event, which is normally required for faithful segregation of chromosomes. Additionally, the average number of COs is relatively low, typically from 1 to 3 events per chromosome (Mercier et al., 2015). In monocentric chromosomes, the density of COs is extremely heterogeneous at both large (chromosomal) and small scales (kb). Peri- and centromeric regions are largely depleted in COs (cold regions) (Petes, 2001; Fernandes et al., 2019). In some extreme cases, such as wheat, up to 80% of the genome hardly ever experience any COs (Choulet et al., 2014). These regions contain ~30% of the genes which are thus out of reach for plant breeding.

To exchange DNA, the chromosomes must undergo double-strand breaks. This process of physiologically induced DNA fragmentation is conserved in the vast majority of eukaryotes and is carried out by the topoisomerase-like protein SPO11 (Keeney et al., 1997; Keeney, 2008). After SPO11 introduces double-strand breaks, the free 3′ ends left are targeted by the recombinases RAD51A and DMC1. These proteins help the 3′ ends to search for homologs as templates for repair. After the invasion of the single strand, a recombination intermediate structure is formed, the displacement loop (D-loop) (Brown and Bishop, 2014). DNA synthesis of both ends generate a new structure called double Holliday Junction (dHJ) (Wyatt and West, 2014). A CO is an outcome of the resolution of a dHJ, but other outcomes are possible (Allers and Lichten, 2001). In this case, the invading strand is ejected from the D-loop and anneal to the single-strand 3'end of the original double-strand break. Crossovers may be resolved in two main ways: the main pathway 1 (exhibiting interference) and a secondary pathway 2 (lacking interference). The pathway 1 is a meiosis-specific process with many associated proteins (the so-called ZMM proteins), namely MSH4, MSH5, MER3, HEI10, ZIP4, SHOC1, PTD (Mercier et al., 2015). This pathway is highly conserved among eukaryotes. The secondary pathway involves the protein MUS81. The existence of additional crossover pathways cannot be excluded (Mercier et al., 2015; Lambing et al., 2017).

### Holocentric Chromosomes

Apart from the monocentric organization, another type of chromosomal organization, the holocentric (holokinetic) chromosomes, evolved independently in many lineages of unicellular eukaryotes, green plants, and metazoans (Melters et al., 2012; Escudero et al., 2016). Holocentric chromosomes have no distinct primary constriction visible while condensed, as they harbor multiple centromeric domains along their lengths (Heckmann et al., 2013; Steiner and Henikoff, 2014; Marques et al., 2015). Thus, spindle fibers attach along almost the entire poleward surface of the chromatids. As a result, sister-chromatids migrate to opposite poles parallel to each other during anaphase, while in the case of monocentric chromosomes microtubule spindles attach to a distinct kinetochore and the sister chromatids move together to opposite poles at anaphase with a clear attachment of microtubules onto the centromere.

Although organisms with holocentric chromosomes are considered relatively rare, clades possessing such chromosomal structure include more than 350,000 species (Kral et al., 2019). Between 1.5 and 2.0% of the flowering plants (~5,500 species) are supposed to have holocentric chromosomes (Bures et al., 2013). Likely, due to the lack of chromosome studies, holocentricity should be even more common than reported.

A multiplication of centromeric sequences from one location to multiple sites along the chromosome arms has been proposed as a possible mechanism of holocentromere formation (Greilhuber, 1995). One common explanation for the evolution of holocentric chromosomes is their putative advantage over monocentric ones when it comes to chromosome breakages and consequent karyotypic variation (Zedek and Bures, 2018). The studies on artificial chromosomal rearrangements in various holocentric species showed that chromosome fragments retaining centromeric activity are stably transmitted during mitosis and meiosis (Heckmann et al., 2014; Jankowska et al., 2015).

Recent findings in holocentrics have brought back the discussion about the chromosome structure plasticity of holocentric lineages, including both CENH3-based and CENH3-less holocentromeres (Marques and Pedrosa-Harand, 2016; Drinnenberg and Akiyoshi, 2017). Such plasticity seems to be evolutionarily advantageous for it would increase the resistance of chromosomes against breaks and fusions. However, no difference in diversification rates between monocentrics and holocentrics seems to occur (Marquez-Corro et al., 2018).

### Meiosis Progression in Holocentric Organisms

The best studied holocentric organism is the animal model *C. elegans*, and much of what we know about meiotic adaptations in organisms with this kind of chromosome structure derives from it (for additional information, see Wormbook (2005). However, due to the independent origin of holocentric organisms, adaptations in distantly related holocentric lineages are likely to be lineage-specific. In *C. elegans*, despite of its unique adaptations, meiosis progress resembles the process in monocentric organisms, in the way that homologs segregate at the end of meiosis I (Lui and Colaiacovo, 2013). During prophase I, chromosome remodeling processes occur, bivalents acquired a cruciform appearance with a long and a short arm and homologs are segregated to opposite poles l in a way similar to canonical meiosis. But the two-step loss of cohesion is accomplished through an alternate mechanism in a LAB-1 (a functional analog of shugoshin) dependent way (De Carvalho et al., 2008).

### Meiotic Progression in Holocentric Plants Is Associated With Inverted Meiosis

Recently, several works have employed modern tools to better characterize the structure and function of holocentric centromeres (holocentromeres) during mitosis and meiosis in plants (Heckmann et al., 2013, 2014; Cabral et al., 2014; Marques et al., 2015, 2016; Oliveira et al., 2019; Neumann et al., 2020). However, the lack of genomic data and functional studies on holocentric plants hamper a better understanding of their cell-division-related adaptations. Upon mitosis, holocentricity does not affect sister chromosome segregation mechanisms, and a parallel migration of sister chromatids substitutes the typical V-shape migration of monocentric chromatids. In contrast, during meiosis several challenges appear because centromeres are not restricted to a single domain as in monocentrics, but rather dispersed across several domains genome-wide.

Thus, the stepwise cohesion release observed in monocentric chromosomes is not possible, since sister-holocentromeres are not associated in holocentric plants precluding their mono-orientation (Cabral et al., 2014; Heckmann et al., 2014; Marques et al., 2016). Additionally, the chromosome remodeling mechanism observed in *C. elegans* is unlike in holocentric plants, since they can have more than one CO per bivalent and maintenance of holocentromeric activity during meiosis forces the bi-orientation of sister-holocentromeres. Therefore, holocentric plants have developed a different kind of meiosis called post-reductional or inverted meiosis to segregate their chromosomes. The phenomenon of inverted meiosis was first reported as early as 1940 in *Carex* (Wahl, 1940) and since then has been found in other holocentric plants of *Cuscuta, Luzula* and *Rhynchospora* (Malheiros et al., 1947; Pazy and Plitman, 1991; Cabral et al., 2014; Heckmann et al., 2014) but also in holocentric insects (Battaglia and Boyes, 1955; Nokkala et al., 2002; Viera et al., 2009). In this type of meiosis, the bivalents align themselves perpendicular to the equatorial plate during metaphase I with bi-orientation of sister-chromatids forcing them to separate to opposite poles during anaphase I (equational division during meiosis I) (Figure 1B). Thus, at the end of meiosis I, the daughter cells remain diploid. During meiosis II, thin chromatin threads are seen connecting the homologous non-sisters, which then separate to the opposite poles (reductional division during meiosis II). Although these chromatin threads are observed in both *Luzula* and *Rhynchospora*, it is not yet known what is the mechanism coordinating these connections (Cabral et al., 2014; Heckmann et al., 2014).

Furthermore, very little is known about the protein dynamics involved in the cohesion release and CO control during inverted meiosis in plants. Besides, in the plant genus *Rhynchospora* (beaksedge) both chiasmatic and achiasmatic inverted meiosis have been observed (Cabral et al., 2014). Apparently, meiotic recombination seems to occur in *R. pubera* (2n = 10), since chiasmata formation and the presence of meiosis-associated proteins (RAD51A, ASY1) have been observed, which represent the normal axis formation and occurrence and processing of DNA double strand breaks. In theory, inverted meiosis should be associated with a complete release of the meiotic cohesin REC8 between sister-chromatids already at end of meiosis I, allowing sisters to segregate at anaphase I (Figure 1B). However, sister-holocentromeres are not associated in holocentric plants, which could potentially interfere with the role of shugoshin. The behavior of cohesin or shugoshin in holocentric plants exhibiting inverted meiosis is unknown. Furthermore, the achiasmatic species *R. tenuis* (2*n* = 4) exhibits no chiasmata (Figure 1C). This species has the smallest reported number of chromosomes in the family and performs meiosis with the formation of four univalents, despite of RAD51 foci being observed, which suggests that DSBs are still occurring but being processed without crossovers (Cabral et al., 2014). Whether a defect in the meiotic machinery of this species is responsible for the achiasmy observed and whether the female meiosis is also achiasmatic is subject to current studies in our group. A similar phenomenon could be identified in a monocentric plant species, *Helianthemum squamatum*, which also exhibits a very small number of chromosomes when compared with close relatives (Aparicio et al., 2019).

**Figure 2:**
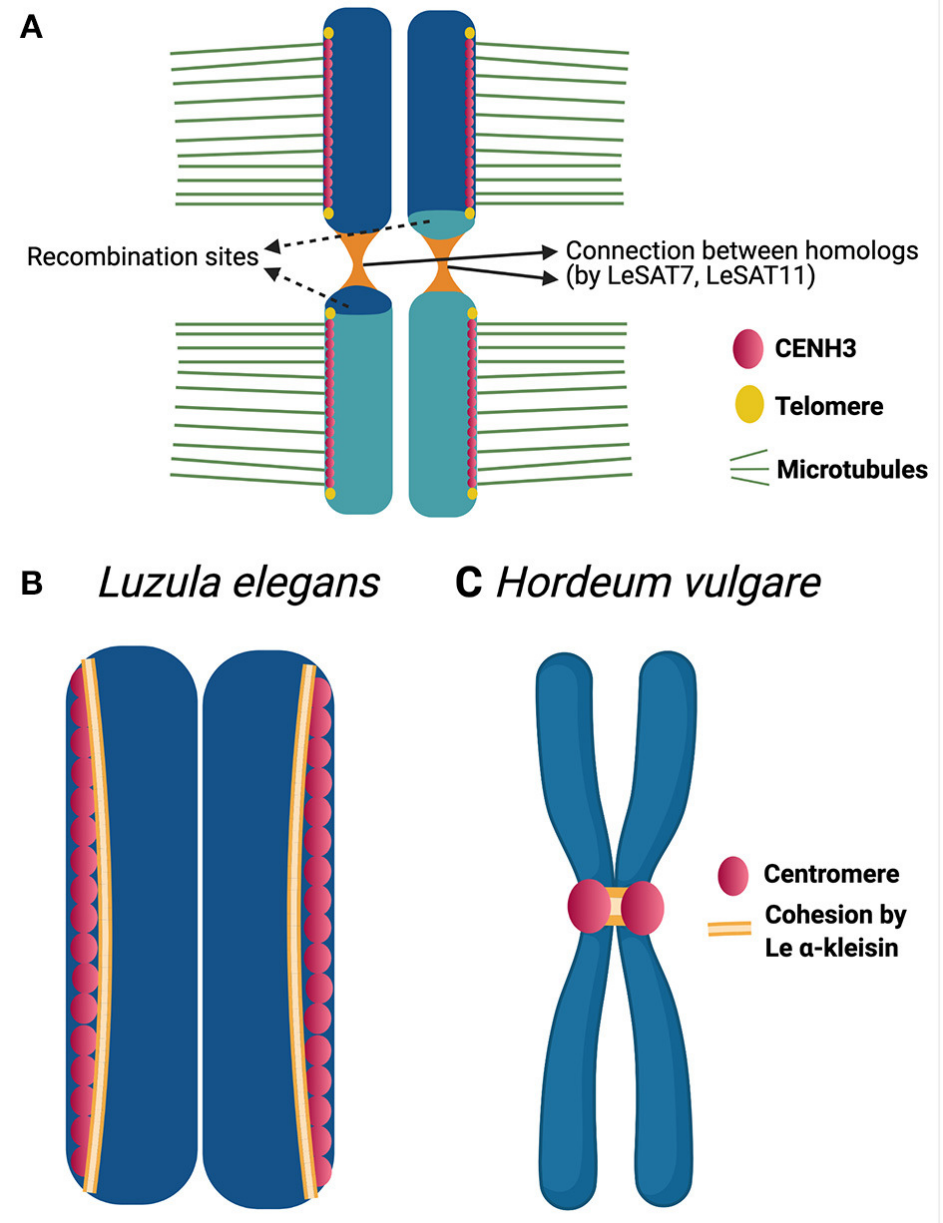
Chromatin threads and cohesion in *Luzula* chromosomes. **(A)** Model highlighting the structural adaptations during inverted meiosis of *Luzula elegans* (see Heckmann et al., 2014) for further details). A rod bivalent with single crossover is illustrated in the model. CENH3 (centromeric protein) appears as a single linear line and centromeres of sisters are not fused. The sister chromatids bi-orient and attach to microtubules from opposite poles. Homologous non-sister chromatids associate with each other by end-to-end connections reported to be established by satellite elements, which maintain non-sister chromatids together up to meiosis II. **(B,C)** CENH3 and Le α-kleisin distribution during mitotic metaphase of *Luzula elegans* **(B)** and *Hordeum vulgare* **(C)** (see Ma et al., 2016) for further details). In the holocentric plant *Luzula*, CENH3(centromeric protein) appear as linear signals during mitotic metaphase. Le α-kleisin appears in the CENH3 regions and not between sister centromeric units. Whereas, in case of the monocentric plant *Hordeum vulgare*, the same Le α-kleisin is reported to present in the centromeric regions as well as establishing a connection between the sister centromeres.

The mechanisms behind the inverted meiosis have been further studied in *Luzula elegans* (Heckmann et al., 2014). Anti-CENH3 immunolabeling patterns appeared as linear lines during mitosis as well as meiosis. The authors propose that, a single linear functional centromere may be formed during meiosis and mitosis. Additionally, CENH3 signals from sisters-chromatids always remain separate. This may help, in the bi-polar orientation of the sisters. Each chromatid makes, end to end connection, by means of thin heterochromatin threads with its homologous partner which starts as early as pachytene. These connections are known to be established by satellite elements like LeSAT7, LeSAT11 and may represent chiasmata preserved at sub-telomeric regions (Figure 2A). A similar hypothesis was also proposed by Ris (1942) while researching on inverted meiosis in aphids. This connection may be involved in ensuring the correct segregation of homologous non-sister chromatids during the second meiotic division. In *Luzula elegans*, immunolocalization with anti-ASY1 and anti-ZYP1 signals were observed as linear lines during early prophase I and telomere bouquet formation was also observed (Heckmann et al., 2014). Thus, early prophase events like DNA double strand break repair, pairing, synapsis and telomere bouquet formation appears the same as canonical meiosis.

Many questions remain a mystery with respect to inverted meiosis. What causes the sisters to separate during meiosis I? Is the cohesion mechanism, which plays a key role in holding the sisters together during meiosis I of canonical meiosis, evolved to enable inverted meiosis? How do the kinetochore proteins assemble and function during inverted meiosis? Monocentric organisms have mechanisms to prevent separase from degrading the cohesion in localized centromeric regions during anaphase I. This enables the sisters to be held together until anaphase II (Nasmyth, 2015). In holocentrics, which have diffuse centromeres, the mechanism of centromeric cohesion protection may be disabled. This may result in the loss of centromeric cohesion and allows the sisters to separate during meiosis I. Attempts to study cohesion mechanism during inverted meiosis were made in *Luzula elegans* (Ma et al., 2016). Signals of LeAlpha-kleisin-1 (cohesin ortholog of AtSYN4) appear during early prophase as reported for cohesin in monocentric meiosis, as demonstrated by immunolabeling. During both mitotic and meiotic metaphases I and II, these signals are observed in CENH3-positive regions but not between sister chromatids (Figure 2B). The authors also carried out the same experiment in the monocentric plant *Hordeum vulgare* (barley). In this experiment using the same antibody, the signals were observed in centromeric regions as well as in between the sister chromatids in mitotic metaphase (Figure 2C). Thus, the cohesion which connects the sister centromeres together in the monocentric species, barley, seems to not play the same role in the holocentric *Luzula*. This may be an early evidence that the function of a cohesin in monocentric may not be the same in a holocentric organism. It is speculated that LeAlpha-kleisin-1 may be involved in the centromere assembly but lost the function of establishing connection between sisters in *Luzula*. We cannot rule out the possibility of other cohesins involved in the connection between sisters. Thus, cohesins as potential candidates to be studied in future may give us more insights.

Anti-CENH3 immunolabeling patterns appeared as linear lines during mitosis in *R. pubera*. However, during meiosis centromeres form clusters (so-called cluster-holocentromeres) along the poleward side of the bivalents where the microtubules attach perpendicularly during meiosis I and the clusters are present in the middle of the chromatids during meiosis II (Marques et al., 2016). Additionally, CENPC, which represents the outer kinetochore protein, is also co-localized with CENH3 in meiosis which may refer to a conserved assembly of meiotic kinetochores on the holocentromeres (Figure 1B). This is the first report about kinetochore proteins in holocentric plants. But still, studies on kinetochore proteins like MIS12 (required for fusion of sister kinetochores), cohesion proteins like SMC1, SMC3, SCC3, REC8 (involved in centromeric cohesion during meiosis I) and shugoshin are necessary to provide more evidence to understand the observed phenomena during inverted meiosis.

The differences in the centromere organization during inverted meiosis of *Luzula* and *Rhynchopsora* show that the mechanisms differ in both cases and the regulation of inverted meiosis may be more complex. Regardless of the differences, in both cases the non-homologous chromatids appear to be connected by thin chromatin threads during meiosis II, as in case of *Luzula* specific tandem repeats were associated to such threads, but the nature of this connection is not yet identified in *Rhynchospora*. Heterochromatic threads seems to play an important role in the separation of achiasmate homologs during female meiosis in *Drosophila* (Hughes and Hawley, 2014). In this particular case the threads seem to be resolved by Topoisomerase II during meiosis I. However, chromatin threads in both *Luzula* and *Rhynchospora* are also observed in meiosis II, whether a similar mechanism occurs in the case of inverted meiosis in these holocentric plants is yet to be shown.

### Meiotic Recombination in Holocentric Organisms

**Figure 3:**
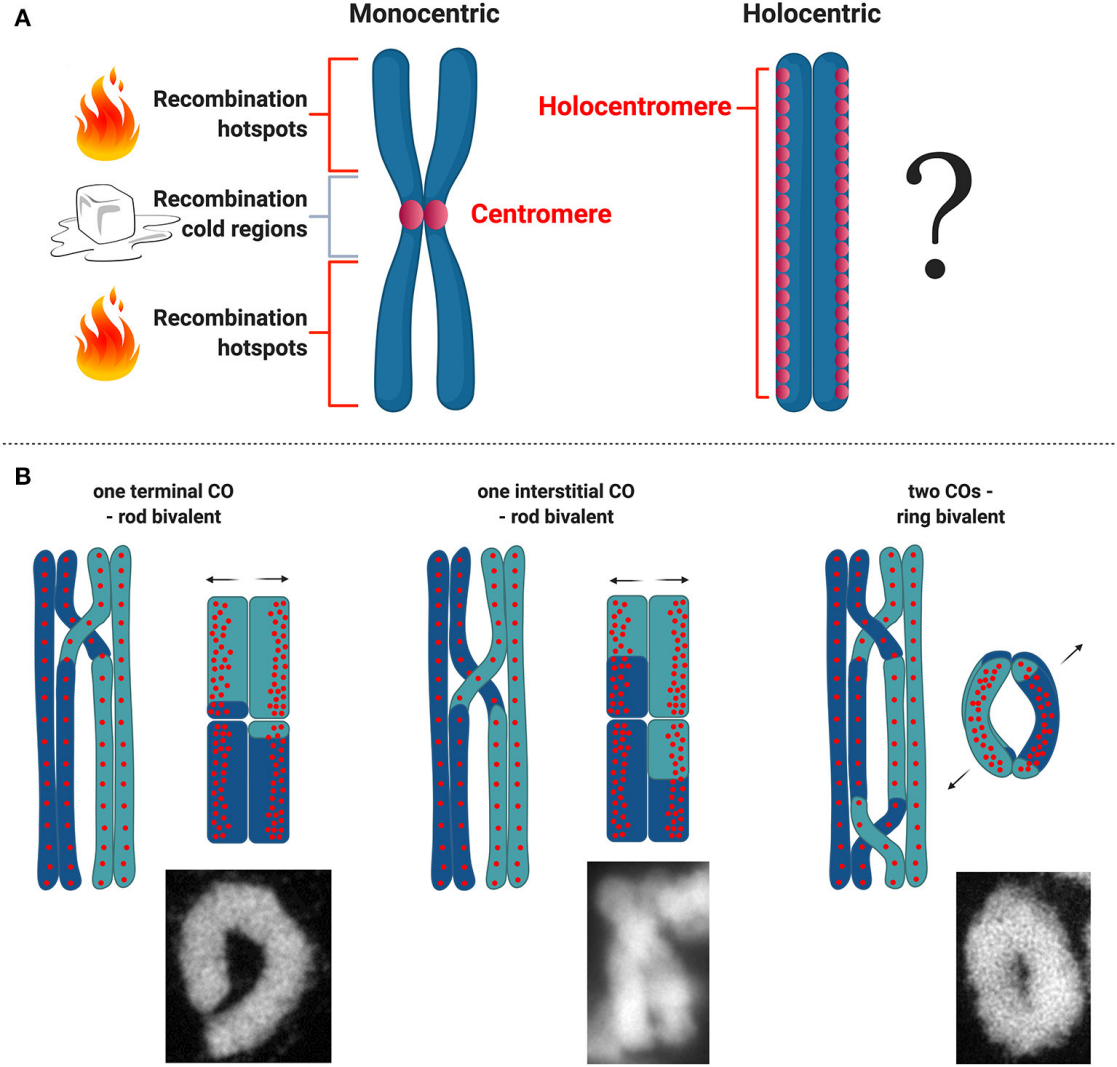
Meiotic recombination in holocentric plants. **(A)** Scheme of what is known about distribution of hotspots for meiotic recombination with respect to centromere organization on monocentric and holocentric plant chromosomes. **(B)** Types of CO and bivalent formation and corresponding models with regard to centromeric units distribution in *R. pubera*. Bivalent microscopic images were made by *M. Castellani*.

Being holocentric can have interesting implications for meiosis. In most eukaryotes and model plant species recombination is suppressed or highly reduced at centromeres (Copenhaver et al., 1998; Fernandes et al., 2019). Recombination at centromeres can disrupt their structural function, impair proper segregation and cause aneuploidy (Nambiar and Smith, 2016). Because of the meiotic recombination suppression at and near centromeres in monocentric organisms, it is of particular interest to understand how meiotic recombination works in organisms with holocentric chromosomes (Figure 3A). However, much of what we know about recombination in a holocentric organism comes from studies in *C. elegans*, wherein centromere proteins such as CENH3 and CENP-C are dispensable during meiosis (Monen et al., 2005) and likely do not affect meiotic recombination. In this case recombination rates broadly vary according to physical position in all six of its chromosomes. Each chromosome is comprised of three large domains: a low-recombining, gene-dense center, and two high-recombining arms (Barnes et al., 1995; Rockman and Kruglyak, 2009).

In Lepidoptera, the largest and most diverse holocentric lineage, meiotic recombination is restricted to male meiosis and frequent karyotype reorganization events are associated with wide variations in chromosome counts across species (Hill et al., 2019). Although high recombination densities were reported for some Lepidopteran insects (Wilfert et al., 2007), this does not seem to be linked to holocentricity.

### Meiotic Recombination in Holocentric Plants

For the time being there are no detailed analysis about recombination frequencies in holocentric plants and all we know derive from basic cytological studies. Recently, the first linkage map for the presumed holocentric plant *Carex scoparia* (Escudero et al., 2018) has been reported, but without the physical map and holocentromere characterization the recombination landscape for a holocentric plant is still unknown. Understanding how recombination is regulated in holocentric plants will potentially unveil new strategies to deal with this chromosome structure during meiosis. Specially in the case of holocentric plants where chromosomes maintain their holocentromere function during meiosis in contrast to *C. elegans* (Heckmann et al., 2014; Marques et al., 2016), which could potentially interfere with the designation of CO events. In the particular case of the plant *R. pubera*, holocentromeres of *R. pubera* extend linearly for the whole length of the chromosomes until their very ends (Cabral et al., 2014; Marques et al., 2015, 2016). Despite the observation that chiasmata frequently link homologs terminally, it seems that recombination in *R. pubera* also happens in internal regions (Figure 3B). Proximity of CO events to centromeric units cannot yet be quantified and recombination may happen in intervals where these units are not present. It is interesting though that centromeric units in *R. pubera* are associated with highly abundant repeats (Tyba repeats), which build short arrays of ~15 kb long and are dispersed genome wide (Marques et al., 2015). In this sense the repeat-based holocentromeres of *R. pubera* seem to assemble in chromatin structures more similar to repeat-based monocentromes. It was estimated that each chromosome should have between 800 and 1,300 repeat-based centromere domains. Taking in account that RAD51 foci are found dispersed in early prophase I (Cabral et al., 2014) and that CENH3 does show similar signals (Marques et al., 2016), DSB sites could potentially occur very close or even within centromeric units.

Cytological observations in *R. pubera* show that at diakinesis five bivalents are present, and physically connected by chiasmata. In this species, ring-shaped bivalents are supposed to be connected by two chiasmata and rod-like bivalents to be connected by only one (Cabral et al., 2014). Observing the shapes of these bivalents, it seems that in *R. pubera* COs are happening mostly at the ends of the chromosomes, but, less frequent, internal COs are also observed. The occurrence of internal COs suggests that recombination events may take place in the vicinity of centromeric repeats (Figure 3B). Similar findings were observed in *Luzula* (Heckmann et al., 2014). Moreover, this is an evidence that the final product of recombination, the crossover, is present at the end of prophase I and that CO interference is occurring as well as CO assurance. Considering the conservation level of the whole ZMM pathway, it seems that meiotic recombination in *R. pubera* is happening and that is not impaired by holocentromeres or inverted meiosis. These observations are quite interesting considering that the holocentromeres in *R. pubera* are repeat-based and distributed along the entire chromosomes in meiosis (Marques et al., 2016). It will be particularly interesting to study whether COs are somehow affected by such centromere distribution and where they are formed.

The molecular basis of recombination repression at centromeres is still not clear. Two possible ways are speculated to happen: either recombination is repressed at the DSB level by modulating the action or the binding of SPO11, or at the level of how DSBs are repaired and processed by the meiosis-specific DMC1 (Nambiar and Smith, 2016). Recent findings using budding and fission yeast has proposed a role for the kinetochore and cohesion as important regulators of DSBs formation within centromeres and surrounding regions (Vincenten et al., 2015; Kuhl and Vader, 2019). Considering the apparent proximity of recombination events and centromeres in *R. pubera*, it is still unclear whether these repression mechanisms exist and if so, how they are regulated. If we look at other well-studied model eukaryotes, the centromere effect appears to be highly conserved and also very efficient in avoiding COs in pericentromeric regions. In *Drosophila melanogaster* the DSB landscape appears to be flat along the chromosome arm, but downstream recombination is then affected by the centromere effect that eliminates pericentromeric recombination intermediates and models the recombination pattern (Hatkevich et al., 2017; Brady et al., 2018). In maize the centromeric effect seems to work with a different mechanism but with the same result. In centromeric regions of maize DSB can be detected, but COs are absent (He et al., 2017). In *Arabidopsis*, Spo11-oligos resulting from Chip-seq experiment are depleted at pericentromeric regions, where CO are also absent, indicating reduced levels of DSBs at these regions (Choi et al., 2018). In yeast, kinetochore complexes protect centromeric regions, reducing dramatically DSB and CO (Vincenten et al., 2015; Kuhl and Vader, 2019).

A similar question involves the presence of so-called hotspots and cold regions of recombination, regions on the chromosomes where recombination is more or less likely to take place. Multiple species, including plants, display hot and cold spots (e.g., centromeric regions) (Choi and Henderson, 2015). However, the presence of holocentromeres in *R. pubera* makes it difficult to predict the presence of hotspots or cold regions or to speculate about their location. Perhaps the situation is that there are no hotspots in *R. pubera* similar to *C. elegans*. A study in *C. elegans* has made a detailed analysis of recombination rate in a 2 Mb region, discovering that there are no clear hotspots, but recombination rates are constant, constrained only by the structural domain of the chromosome arm (Kaur and Rockman, 2014). This is a unique case similar only to S. *pombe*, which is not holocentric.

A different case is the one of the holocentric relative *Rhynchospora tenuis*. In this species chiasmata are not observed and at least male meiosis seems to be complete achiasmatic (Figure 1C). The further observation of RAD51 during early prophase I suggests, in principle, that DSBs are being formed (Cabral et al., 2014). The absence of recombination outcomes might be evidence of the disruption of the ZMM recombination pathway in one or more points. Mutations in the SC of *C. elegans* negatively affect recombination and crossover regulation (Colaiacovo et al., 2003). However, this behavior is not consistent among plant species. For instance, in barley it was reported that dramatic reduction of normal levels of ZYP1 by RNAi also drastically reduce CO formation (Barakate et al., 2014). However, in the case of *Arabidopsis* and rice a malfunctioning SC does not affect recombination and may even increase CO frequency and abolish CO interference (Wang et al., 2010; Capilla-Perez et al., 2021; France et al., 2021). In both holocentric *Rhynchospora* and *Luzula* it was shown that they apparently have conserved SC structures as immunostaining with SC proteins showed the conserved pattern for monocentric species (Cabral et al., 2014; Heckmann et al., 2014). Whether SC proteins are involved in CO regulation in holocentric plants is currently unknown and should be subject of future studies.

An interesting point in holocentric clades is that chromosome numbers tend to vary greatly within the group which could be a consequence of lack of centromere constrain. However, this may not be true for all holocentric clades (Ruckman et al., 2020). In the *Cyperaceae* family, which is the largest group of holocentric plants, chromosomes vary from *n* = 2 to *n* = 108 (Roalson, 2008). Although the lowest chromosome number in angiosperms is found in *Rhynchospora tenuis* (*n* = 2), we can also find extraordinarily very high chromosomes numbers in other genera within this family, for instance in *Carex* (Wieclaw et al., 2020) and *Cyperus* (Roalson, 2008). Since the number of chromosomes is proportional to recombination rates, high chromosome numbers would also impose higher recombination rates in holocentric plants, specially, in this case where the number of chiasmata tends to be typically low, with one or two CO per bivalent. However, a fitness balance must exist otherwise holocentric organisms would tend to have always high chromosome numbers, which is not the case. High chromosome numbers would potentially increase the complexity of the recombination process with likely more possibilities of mistakes in the segregation process.

### Holokinetic Drive

Besides the occurrence of inverted meiosis, holocentric sedges (Cyperaceae) also exhibit another peculiar process: the formation of pseudomonads by the end of the microspore meiosis (Rocha et al., 2016, 2018). During this process, three microspores degenerate and only one proceeds with gametogenesis. Thus, only one pollen grain results from each meiotic event in these plants. This specific feature could relate the segregation process with the size of the chromosomes in a process called holokinetic drive, which was first introduced by Bures and Zedek (2014). According to this hypothesis, there would be a selection for chromosomal size upon meiosis. Either the smallest or the largest chromosomes would be favored depending on the case, and formation of pseudomonads could accelerate this process. A negative correlation between chromosome number and total genome size observed in several holocentric groups seems to support this. For instance, this correlation has been recently reported for the genus *Rhynchospora* (Burchardt et al., 2020). Moreover, it has been recently proposed that centromere drive could occur in association with holokinetic drive in members of *Cyperaceae* and, thus, the meiotic asymmetry in both sexes of this family could increase the potential for selfish centromeres to gain an advantage in both male and female meiosis (Krátká et al., 2021). Alternatively, the selection of the survival cell could be related with the results of the recombination process, wherein the best combination of alleles resulting from the meiotic event would be selected.

## Perspectives and Future Aims

The mechanisms behind inverted meiosis in holocentric organisms are currently unknown. The occurrence of inverted meiosis demands modification in the conserved mechanisms of meiotic cohesion and chromosome segregation. New adaptations and differential regulation of meiotic cohesions such as REC8 and centromere cohesion guardians such as shugoshins are expected to have happened. Additionally, modification of the spindle attachment machinery also should be expected due to an alternative centromeric organization. Furthermore, the observed chiasmata formation between holocentric chromosomes demands adaptations of the mechanisms that prevent recombination at or around centromeres. The limited knowledge of holocentromeres and close relatives of *Cyperaceae* limits us to speculate about what to expect in terms of adaptations of the meiotic recombination machinery to holocentricity. Future studies aiming the molecular characterization of such mechanisms will be of interest for evolutionary and comparative biology studies.

## Author Contributions

PH drafted the section about the meiotic machinery in eukaryotes, incorporated the different contributions and reviewed the text. GT drafted the sections about meiosis progression and inverted meiosis in holocentrics. MC drafted the section about meiotic recombination. AM drafted the sections about holocentric plants, reviewed and supervised the production process of the manuscript. Figures were made by GT and AM. All authors contributed to the article and approved the submitted version.

## Conflict of Interest

The authors declare that the research was conducted in the absence of any commercial or financial relationships that could be construed as a potential conflict of interest.
